# Oceanic Sharks Clean at Coastal Seamount

**DOI:** 10.1371/journal.pone.0014755

**Published:** 2011-03-14

**Authors:** Simon P. Oliver, Nigel E. Hussey, John R. Turner, Alison J. Beckett

**Affiliations:** 1 School of Ocean Sciences, University of Wales, Bangor, Menai Bridge, Anglesey, Wales, United Kingdom; 2 The Thresher Shark Research and Conservation Project, Malapascua Island, Cebu, The Philippines; 3 Great Lakes Institute for Environmental Research, University of Windsor, Windsor, Ontario, Canada; 4 School of Biomedical Sciences, Nuffield BD, University of Liverpool, Liverpool, United Kingdom; National Oceanic and Atmospheric Administration/National Marine Fisheries Service/Southwest Fisheries Science Center, United States of America

## Abstract

Interactions between pelagic thresher sharks (*Alopias pelagicus*) and cleaner wrasse were investigated at a seamount in the Philippines. Cleaning associations between sharks and teleosts are poorly understood, but the observable interactions seen at this site may explain why these mainly oceanic sharks regularly venture into shallow coastal waters where they are vulnerable to disturbance from human activity. From 1,230 hours of observations recorded by remote video camera between July 2005 and December 2009, 97 cleaner-thresher shark events were analyzed, 19 of which were interrupted. Observations of pelagic thresher sharks interacting with cleaners at the seamount were recorded at all times of day but their frequency declined gradually from morning until evening. Cleaners showed preferences for foraging on specific areas of a thresher shark's body. For all events combined, cleaners were observed to conduct 2,757 inspections, of which 33.9% took place on the shark's pelvis, 23.3% on the pectoral fins, 22.3% on the caudal fin, 8.6% on the body, 8.3% on the head, 2.1% on the dorsal fin, and 1.5% on the gills respectively. Cleaners did not preferentially inspect thresher sharks by time of day or by shark sex, but there was a direct correlation between the amount of time a thresher shark spent at a cleaning station and the number of inspections it received. Thresher shark clients modified their behavior by “circular-stance-swimming,” presumably to facilitate cleaner inspections. The cleaner-thresher shark association reflected some of the known behavioral trends in the cleaner-reef teleost system since cleaners appeared to forage selectively on shark clients. Evidence is mounting that in addition to acting as social refuges and foraging grounds for large visiting marine predators, seamounts may also support pelagic ecology by functioning as cleaning stations for oceanic sharks and rays.

## Introduction

Seamounts are hotspots of biodiversity in the open ocean [1]-[3]. They also act as stepping-stones from which marine species spawn and dispense their larvae [1], [4], and have been identified as important habitat for large visiting marine vertebrates [3], [5]. Although the ecological significance of seamounts attracting elasmobranchs is poorly understood, it has been suggested that they function as daytime social refuges for nocturnally foraging sharks, which navigate to and from them by using signature intensities of geomagnetic fields and topographical features [6]. Here, we show that cleaner wrasse on seamounts service visiting pelagic thresher sharks (*Alopias pelagicus*).

The pelagic thresher shark is one of three recognized thresher shark (*Alopiidae*) species [7]. The shark reaches 365 cm in length, half of which comprises an elongate tail fin [7]. Known from fisheries [8] and by-catch to frequent warm and temperate offshore waters circumglobally [9], pelagic thresher sharks mature late, have low fecundity and are classed as *Vulnerable* by the International Union for the Conservation of Nature and Natural Resources' (IUCN) Red List [10]. For the past two decades, pelagic thresher sharks have been observed by SCUBA divers to visit Monad Shoal, a shallow coastal seamount in the Philippines, where they interact with cleaner fish *Labroides dimidiatus* and *Thalassoma lunare*. Sharks, including pelagic thresher sharks, host a variety of parasites (Oliver current data) [11], and it is proposed that they visit cleaning stations at this site to control infection [12].

Sharks infected with ectoparasites suffer a variety of health consequences, which may include anaemia [13], the retarded development of reproductive organs [14], reduced respiratory efficiency [15], [16], and chronic and debilitating skin disease. Severe infections in captive sharks have been known to catalyse behavioral modifications such as flashing and rubbing against the sides and substratum of aquaria, and interacting with cleaner fish [17].

Cleaning mutualisms within coral reef communities are well documented [18], [19]. Small fish or shrimps termed ‘cleaners’ forage on ectoparasites, tissue and mucus from larger ‘client’ reef fish. The blue streaked cleaner wrasse (*Labroides dimidiatus*) is among the most studied of the 130 described marine cleaner species [19]. They occupy small territories known as ‘cleaning stations’ that reef fish clients visit for ‘cleaning services’. Clients may ‘pose’ by head or tail standing to solicit a cleaner to inspect them, or the inspection may take place without a solicitation [20]. Parasite infestation may be the most likely cue for clients seeking cleaners [21], [22]. Cleaners appear to control the parasite loads of their clients [12], but there is less evidence to show that their services have a positive effect on client health or reproductive success [23].

According to optimal foraging theory, an individual should forage more on a food patch where food is plentiful and profitability is maximized by energy reward in relation to search and handling time [24]. When investigating how cleaners forage, Bshary and Grutter [25] showed that *L. dimidiatus* spent more time on, and took more feeding bites from the parasitized side of the surgeonfish *Ctenochaetus striatus* compared to the unparasitized side. They concluded that cleaners optimize their foraging by concentrating on areas of a client's body where parasites are located. Foraging by cleaners on clients should therefore be dependent on the quality of the food patch, and the relative ease with which food may be obtained. Since ectoparasite distributions on sharks are typically site specific [11], [16], [26], it can be predicted that cleaners will forage most at these locations.

In this paper, we provide evidence to show that a pelagic shark species visits a seamount where it interacts with cleaners and investigate the cleaner-client association. Behavioral interactions were quantified between pelagic thresher sharks and cleaner wrasse from remote video observations to address the following hypotheses: (1) cleaners selectively forage on specific areas of thresher shark clients; and (2) thresher shark clients modify their behavior to facilitate inspections from cleaners. The cleaner-thresher shark association is discussed in comparison to known trends in the cleaner-reef teleost system.

## Methods

### Location

**Figure 1:**
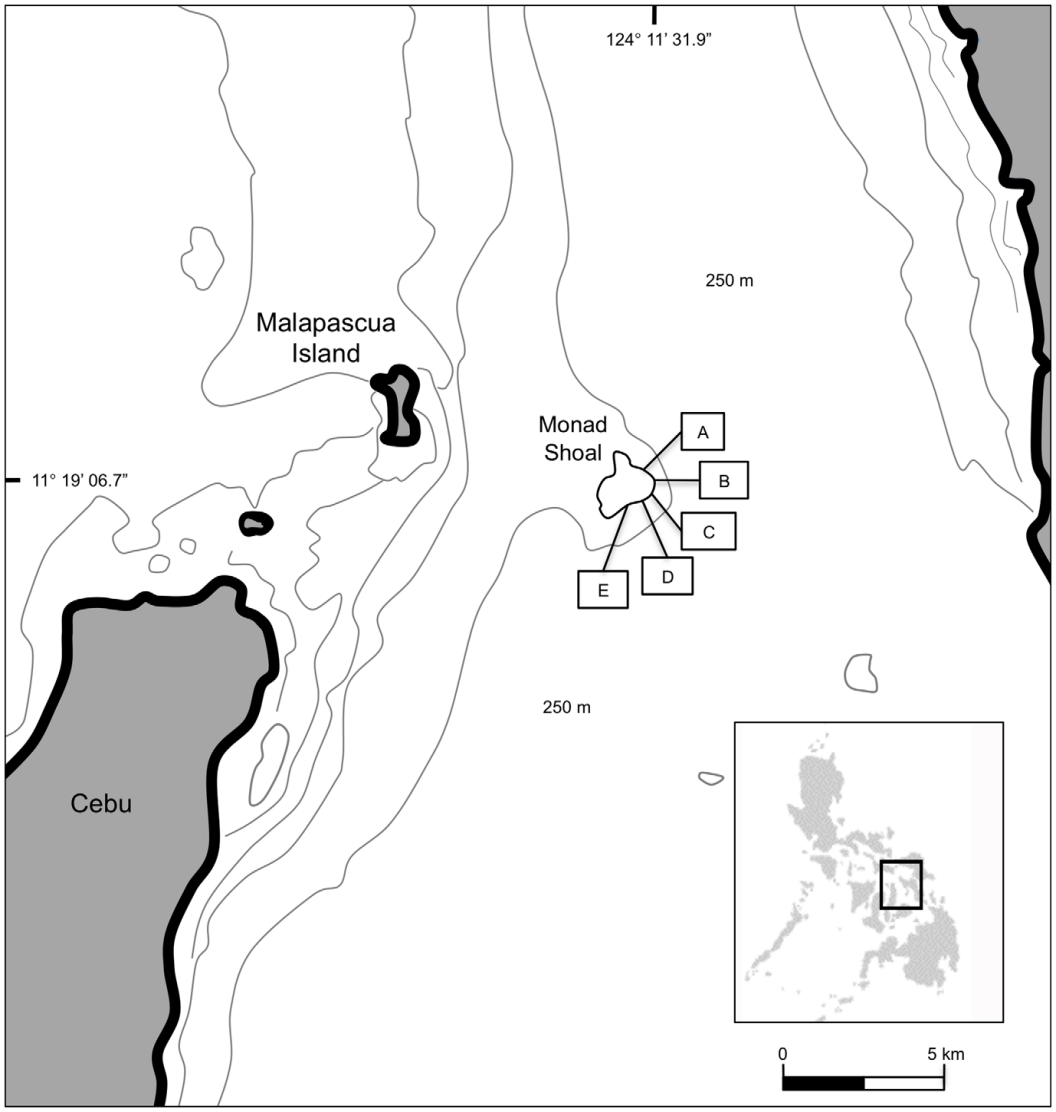
Map showing the location of Monad Shoal off Malapascua Island, the Philippines. Five cleaning stations were identified (A–E) approximately 100 m apart on the southeast section of the plateau.

Monad Shoal is a seamount rising 250 m from the sea floor in the Visayan Sea (N 11° 19′ 06.7″, E 124° 11′ 31.9″), eight kilometers due east from Malapascua Island, Cebu, in the Philippines (Figure 1). The top of the mount forms a plateau at 15 to 25 m depth, with a surface area of 4.5 km^2^. The low profile *Acropora* coral community is now degraded and dominated by rubble, caused by decades of dynamite fishing. Recreational divers visit the seamount to observe thresher sharks on most days, and dive tourism generates important income for the region.

Five cleaning stations (identified here as A–E) were identified approximately 100 m apart on the southeast section of the plateau (Figure 1).

### Sampling

A remote video camera was deployed using SCUBA at one of the five cleaning stations between 06:00 and 16:00 hours on 232 days over 16 months (between July 2005 and December 2009). During the first field phase (July – September, 2005) the deployment station (A–E) was selected *in situ* by directly observing which station had cleaners present, which were actively signaling for clients or inspecting reef teleosts (cleaners were not counted due to time constraints inherent with SCUBA diving). Based on observations from the first field phase, station A was favored for subsequent phases since recordings of thresher sharks interacting with cleaners were most regular there.

From July to September 2005, a Sony Handycam® (DCR-PC 330) preset to record for 90 continuous minutes with focal range locked to 0.6 m was used. To lengthen the duration of observations, the remote video camera was upgraded to a Sony Handycam® HDR-SR8 preset to record for 360 continuous minutes with focal range locked to 0.3 m, for subsequent field phases. Underwater housings (Amphibico *Prowler* and *Elite*) fitted with a 100° wide-angle lens were used for all camera deployments. Deployments took place between 06:00 and 16:00 hours, with start times dependent on field conditions. The camera was retrieved at the end of each deployment period. Data were downloaded to a hard drive and footage screened for observations of pelagic thresher sharks.

The time dive boats arrived on site and recreational SCUBA divers entered the water were recorded *in situ* on all camera deployment days. It was assumed that: (i) divers entered the water within 15 minutes of arriving on site; (ii) divers stayed submerged for between 10 and 70 minutes; and (iii) boats departed from the site within 120 minutes of their arrival. Due to the large numbers of divers entering and exiting the water from different boats, only diver entry and boat arrival times were recorded. The mean numbers of divers and boats presented are therefore conservative with some overlap between time intervals inevitable (Table S1).

### Analysis of Video Recordings

Video sequences documenting interactions between thresher sharks and cleaner wrasse on the cleaning station were classified into two main event types: those which (1) resulted in cleaning interactions, or resulted in cleaning interactions that were interrupted; or (2) did not result in cleaning interactions. Events that resulted in cleaning interactions with or without interruption (Type One) typically took place over several minutes and involved inspections (so termed for the appearance of a cleaner to approach and ‘inspect’ a client) made of the same shark client(s) by the same cleaners. Interruptions of cleaning interactions were generally caused by the arrival of other large elasmobranch clients or SCUBA divers. Clients, which swam directly through a station without returning into view, characterized events, which did not result in cleaning interactions (Type Two). These were termed ‘pass’. Events began at the time a thresher shark first entered into view (with no shark presence > five minutes prior) and ended when it exited the station and was no longer in view (>five minutes post).

In a total of 1,230 hours of remote video deployment, 97 events resulting in cleaning interactions were recorded (Type One), 19 of which were interrupted. A total of 20 passes (Type Two) were also recorded.

### Analysis of Thresher Shark Behavior

Events were divided into segments, each of which comprised either one ‘swim circle’ or a pass. Segments were analyzed in 29 frames s^−1^ resolution using *Adobe-Premiere Pro (CS4)* to document behavioral interactions and construct ethograms [27]. Shark sex was determined by the presence or absence of claspers. Examples of the video data are available in the supporting information (Videos S1, S2, S3, S4, S5, and S6).

### Classifying Behaviors

**Figure 2:**
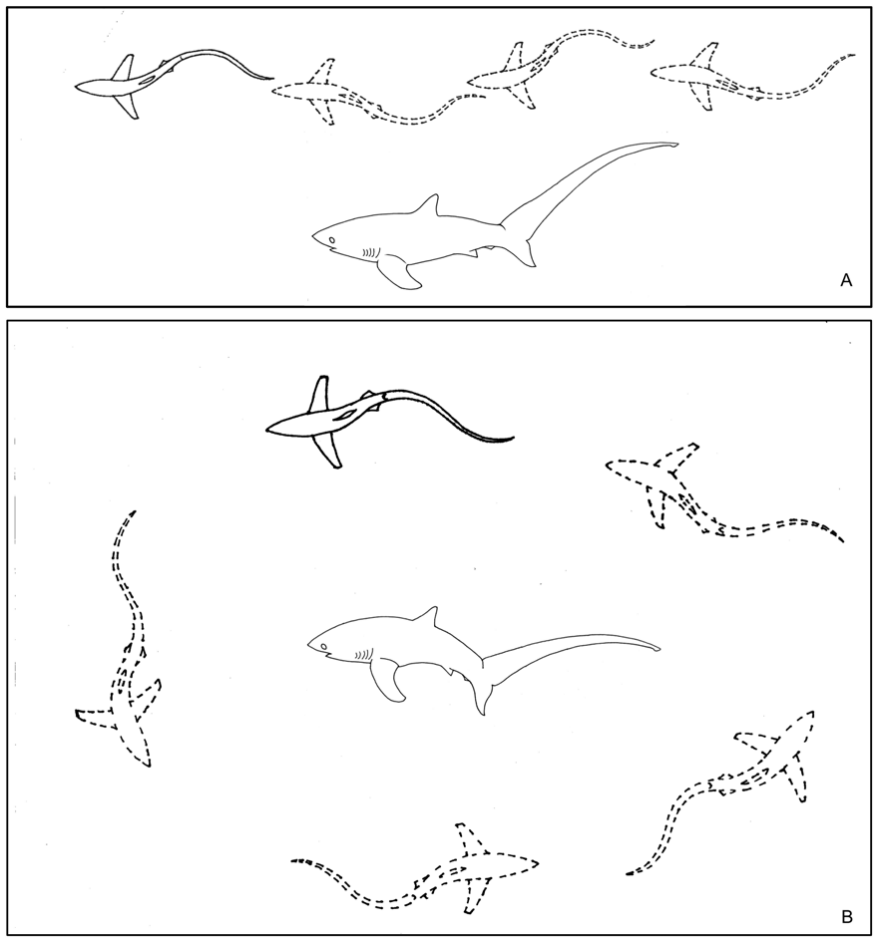
Ethograms of pelagic thresher shark pass and pose behaviors. A) Represents the swimming behavior in Type Two events termed *pass.* These events did not result in cleaning interactions and were characterized by the shark swimming in a straight line at a rate of 37.7±5.1 video frames per body length. B) Represents the swimming behavior in Type One events termed ‘circular-stance-swimming’. These events always resulted in cleaning interactions and were characterized by the shark lowering its caudal fin and swimming in a circular direction at a rate of 60.34±7.55 video frames per body length.

Slater's protocols for categorizing behavior [28] were used to differentiate the behavioral patterns observed in thresher sharks as they interacted with cleaners. Swim speeds, direction of locomotion and posing patterns were used to compare behaviors between the event types (Figure 2). To assess the differences between the relative swim speeds of cleaning (Type One) and passing (Type Two) thresher sharks, the mean number of video still frames used to travel one body length were compared (Table 1). Since Type One (n = 97) and Two (n = 20) events did not occur equally, five subsets from 20 randomly selected Type One events were sampled for uniformity. Video still frames were only sampled when the shark was positioned at a straight angle, perpendicular to the camera.

**Table 1:** Thresher shark swim speeds for Type One and Type Two events.

	*Mean (± sd)*	*Min*	*Max*	*n*
**Pass**	37.70±5.1	28	47	20
**Circular-Stance-Swimming**	60.34±7.55	42	87	20

Means of the number of video frames used by pelagic thresher sharks to travel one body length ± standard deviations are presented along with the lowest (min) and highest (max) counts per event type. n represents the number of events sampled for each event type.

### Food Patches and Cleaner Inspections

To test whether cleaners forage on specific areas of a thresher shark's body, it was assumed that site-specific parasite infections represent high quality food patches. Areas of a shark's body known to harbor concentrations of parasites [11], [14]-[17], [26] were adapted and marked on a photograph of a pelagic thresher shark. These were termed ‘patches’ and categorized as ‘body’, ‘caudal fin’, ‘dorsal fin’, ‘gills’, ‘head’, ‘pectoral fins’ and ‘pelvis’. The pectoral patch included both pectoral fins and the pelvis comprised the cloaca, both pelvic fins and the anal fin.

Patch surface areas were calculated as the proportion of pixels each patch occupied of the photograph relative to the number of pixels occupied by the total body area of the shark, using an image histogram in *Photoshop CS4* (Adobe, San Jose, CA). Proportional patch areas were defined as: body 0.43, caudal 0.15, dorsal 0.03, gill 0.02, head 0.23, pectoral 0.09 and pelvis 0.04.

Cleaner inspections recorded during event segments were marked onto the patches. Because it was not possible to tell from video recordings whether inspections resulted in feeding on parasites, they were used as a proxy for foraging and cleaners were not separated by species.

### Statistical Analysis

All statistical analyses were carried out in *GenStat 8.1* (Rothamsted Experimental Station, Harpenden, UK) and *Minitab 15* (Minitab Inc., State College, PA, USA).

**Figure 3:**
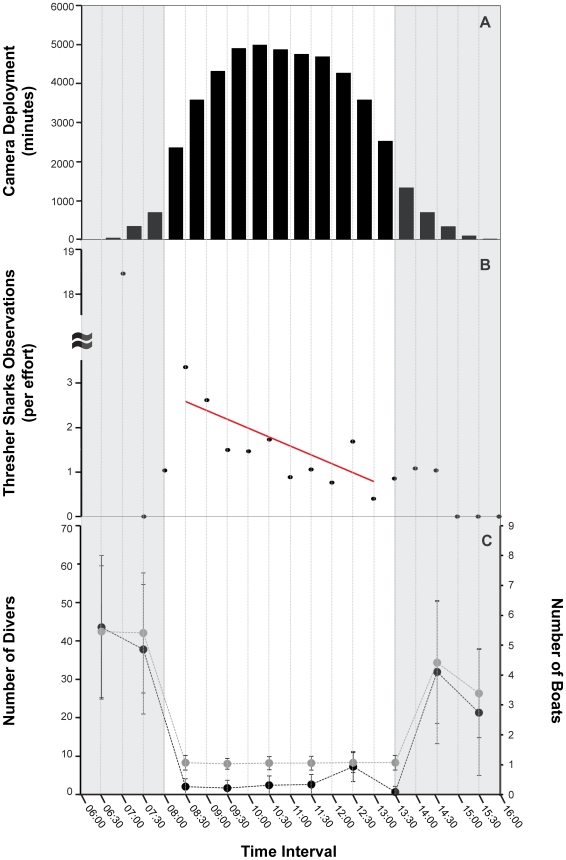
Overall sampling effort during 198 days of field observations from April 2008 to December 2009, by time of day. A) Histogram of the total time the remote video camera was deployed (in minutes), by 30-minute time of day intervals. B) Number of recorded thresher shark events, which resulted in cleaning interactions per camera deployment effort, by 30-minute time of day intervals. C) Mean ± standard deviations of the number of recreational divers (black points) and dive boats (grey points) that visited Monad Shoal during all field phases, by 60-minute time of day intervals. Shaded areas indicate time intervals where the total camera deployment time was <2000 minutes. These were not included in the regression analysis.

Least squares linear regression analysis was used to examine the frequency of interactions between pelagic thresher sharks and cleaners by time of day. Prior to analysis, data on shark observations between 06:00 and 16:00 hours were standardized by dividing the number of sharks observed by the total camera deployment time (per 30 minute time interval), and expressed as number of sharks, per time interval, per 720 minutes (due to small frequency values for shorter time blocks, a 12-hour block was selected for analysis). Because camera deployment times were unevenly distributed across the 30 minute intervals (Figure 3-A), only data between 8:00 and 13:30 hours, where the total deployment time was >2000 minutes, were included in the regression analysis (Figure 3-B).

To investigate whether cleaner inspections varied by sex of shark, a one-way analysis of variance (with number of inspections as response variables and sex of shark as treatment) was employed. All data were log_10_ transformed prior to analysis for normalization and to generate conservative significance values. To assess if there was a relationship between (i) the number of cleaner inspections and (ii) total event time by time of day, least squares linear regression analysis was used. Time of day was defined as the start time of the cleaning event considered in analysis.

To test whether cleaner fish selectively inspected areas of thresher sharks, a log linear model based on a Poisson distribution was used to compare inspections recorded per patch, against the null hypothesis that expected inspections were uniformly distributed across the patches [29], [30].

A saturated model was formulated which included all factors assumed to be exerting influence on observed inspection distributions (observed cleaning events (n = 97) and the defined patches (n = 7)). A series of models controlled for factors in a linear fashion against the saturated design. Accumulated deviances arising from the control effect or interaction were derived, and the goodness of fit, measured by the likelihood ratio (deviances were tested against Chi square) was calculated. The log linear model was used to control for the effects of the covariates: patch (Model 1) and sex (Model 2). Finally, an offset was included (Model 3) to account for variable patch sizes. This enabled the prediction of the expected proportion of cleaner inspections per patch area against the null hypothesis that the proportion of expected inspections was proportional to patch surface area.

If the number of inspections on shark (j) and patch (i) was n_ij_, then the expected number of inspections was µ_ij_:
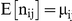



The log linear model for expected cleaner inspections per patch was defined as:
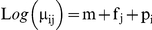
where (m) was the intercept, (f) effect of shark, and (p) the estimated proportions of inspections (with shark 1, and Body patch being factor reference levels).

The expected (exp) relative numbers of cleaner fish inspections on patch i relative to p_1_ was calculated from exp(p_i_)/exp(p_1_)  =  exp(p_i_ – p_1_).

The difference in relative frequencies of inspections on different patches was estimated using p_i_. The log (p_i_/p_1_) was used to calculate the expected proportions of inspections on each patch (exp(p_i_)/exp(p_1_)  =  exp(p_i_)/Σexp(p_i_)).

In the offset model (Model 3), expected cleaner inspections per actual patch area were included by substituting term (µ_ij_) by (µ_ij_/h), where (h) was the actual patch area.

To test whether cleaners showed preferences for specific patches, a *post hoc* analysis (Students t test) used pairwise comparisons between the estimated proportions of inspections per patch (log(pi/p1)) for the patch surface areas (log(hi/h1)).

It was not possible to identify individual animals, therefore the true number of independent observations of sharks may be less than the total number of observations recorded. For example, a shark, recognized by an injury to its left pectoral fin, returned to the site on more than one occasion. Further, the number of cleaning inspections may be underestimated, because cleaner fish activity behind a shark could not be observed on video recordings. The results were therefore interpreted conservatively and all statistical tests were deemed significant at α = 0.01.

## Results

### Recorded Events

A total of 97 events, which resulted in interactions between cleaners and thresher sharks (Type One), were recorded overall (2005–2009). Since camera deployment times were only compiled for field legs spanning 2008–2009, the four events recorded in 2005 were dropped for effort related analysis (n = 93). The mean (±SE) camera deployment time per 30-minute interval between 08:00 and 13:30 hours was 3,848±346 minutes (Figure 3-A).

Observations of cleaning events were recorded at all times of day but their frequency declined gradually from morning until evening (f_1,10_ = 13.55, r^2^ = 0.53, p<0.004) (Figure 3-B). There was no significant effect of time of day on either the number of cleaner inspections (f_1,76_ = 0.13, p = 0.718) or the total event time (f_1,69_ = 0.24, p = 0.627).

There was a significant correlation between total event duration and the number of inspections conducted by cleaners on thresher shark clients (r_76_ = 0.694, p<0.0001, n = 2,757). The mean (±SE) event duration was 6.27±0.53 minutes (95% CI: 5.22–7.32 minutes). The longest recorded event lasted 23 minutes and comprised 210 cleaner inspections; the shortest lasted one minute and comprised three cleaner inspections.

Of the 97 events, which resulted in cleaning interactions, 78 were uninterrupted (Video S1) and 19 were interrupted. When considering only uninterrupted events in which thresher shark clients could be distinguished by sex (n = 71), a one-way ANOVA found no significant difference between the number of cleaner inspections by sex of shark (f_1,69_ = 0.03, p = 0.863).

Among the 19 interrupted events, 12 involved the arrival of a second elasmobranch and were characterized by two clients interacting with the same cleaners over the same station at the same time. Seven of these involved intraspecific interactions between two thresher sharks (Video S2), and the remaining five involved interspecific interactions between different elasmobranch species (two by grey reef sharks, two by manta rays and one by a devil ray)(Videos S3, S4 and S5). The rest of the interrupted events were influenced by the arrival of SCUBA divers (Video S6).

### Distribution of Inspections

**Figure 4:**
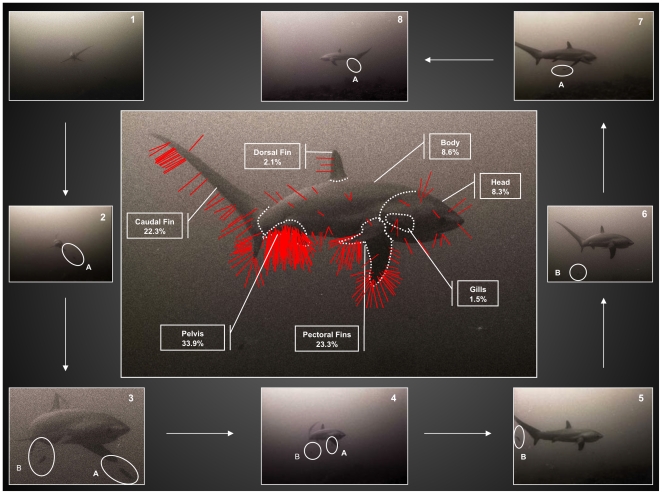
Observed distributions of cleaner fish inspections of pelagic thresher shark clients in 1,230 hours of remote video on 232 days over 16 months (July 2005 to December 2009) (center), with ethogram (around). Red lines represent cleaner inspections (n = 2,757) and were mapped according to their respective locations on pelagic thresher shark clients as observed from remote video. One line represents 20 inspections. 1) A pelagic thresher shark decelerated to 60.34±7.55 video frames per body length 2) a cleaner (A) rose to the pectoral fins 3) another cleaner (B) rose to the pelvis as the former (A) inspected the left pectoral fin 4) pectoral (A) and pelvis inspections (B) 5) caudal inspection (B) 6) cleaner (B) returned to the substrate 7) pectoral inspection (A) 8) cleaner (A) returned to the substrate.

A total of 2,757 cleaner inspections were observed from video records for all cleaner-thresher shark events combined, but their distribution was uneven, with most occurring on the pelvis (33.9%), the pectoral (23.3%) and caudal fins (22.3%), and least on the dorsal fin (2.1%) and the gills (1.5%) (Figure 4).

The first fit of the log linear model (Model 1) found that patch contributed the greatest effect (LRT_6_ = 1891.405, mean deviance >1; p<0.0001), but a large proportion of the residual variance remained unexplained (Table 2).

**Table 2:** Log linear model (Model 1) for the number of cleaner inspections controlling for the effect of individual shark and patch.

*Function*	*df*	*Deviance*	*Mean Deviance*	*Significance*
**+ Shark**	96	2288.767	23.841	<0.0001*
**+ Patch**	6	1891.405	315.234	<0.0001*
**Residual**	576	811.506	1.409	
**Total**	678	4991.678	7.362	

Model 2 found that shark sex (LRT_12_ = 35.893, p = 0.0003) had only a small effect (mean deviance ∼1) relative to patch effect (Table 3).

**Table 3:** Log linear model (Model 2) for the number of cleaner inspections controlling for the effects of patch and shark sex.

*Function*	*df*	*Deviance*	*Mean Deviance*	*Significance*
**+ Patch**	102	4180.172	40.982	<0.0001*
**+ Patch × Sex**	12	35.893	2.991	0.0003*
**Residual**	552	726.721	1.317	
**Total**	678	4991.678	7.362	

The offset (Model 3) demonstrated that the estimated proportion of cleaner inspections was not dependent on patch area. Proportional patch area explained the difference between model 3 and the null model (LRT_102_ = 4180.172, p<0.0001) (Table 4).

**Table 4:** Log linear model (Model 3) with patch area included as an offset.

*Function*	*df*	*Deviance*	*Mean Deviance*	*Significance*
**+ Shark**	96	2288.767	23.841	<0.0001*
**+ Patch Area**	6	4345.081	724.180	<0.0001*
**Residual**	576	811.506	1.409	
**Total**	678	7445.353	10.981	

*Post hoc* comparisons (Table S2) showed that there were no significant differences among the distributions of cleaner inspections between the dorsal and gill patches, or the body and head. There were also no significant differences between the head and the dorsal fin, the body and the dorsal fin, or the head and gills. However, there was a significant difference between the caudal and pectoral fins (p = 0.005), and all other pairwise comparisons were highly significant (p<0.0001). Significant differences between the pelvis and all other patches were also evident (p<0.0001). Patches were ranked (according to cleaner preferences) as highly preferred (pelvis), preferred (pectoral and caudal fins), less preferred (body, head) and not preferred (dorsal fin and gills). Test scores, significance values and confidence intervals are presented in Table S2.

### Circular-Stance-Swimming

A student's T-test showed that during their interactions with cleaners (Type One events), pelagic thresher sharks swam at slower speeds than during passes (Type Two events) (t_19_ = 33.06, p<0.001) (Table 1). Pelagic thresher sharks also lowered their caudal fin in a pose as they systematically circled over cleaner territories (Figure 2-B). This behavioral sequence, which was consistent and repetitive, was categorized as ‘circular-stance-swimming’ (Figure 4, Videos S1, S2, S3, S4, S5 and S6).

## Discussion

While the cleaner-reef teleost system has received considerable attention, little is known about cleaner associations with elasmobranch clients. This study represents the first attempt to quantify interactions between cleaners and oceanic sharks in their natural environments, and underpins the ecological importance cleaning services may play in structuring marine communities, which visit seamounts.

### Recorded Events

The results corroborate local anecdotal evidence that observations of pelagic thresher sharks interacting with cleaners occur on the seamount at all times of the day but that their frequency is greater during early daylight hours. Thresher sharks swim continuously and are nocturnally active [7], [31], [32], therefore the mechanisms of parasite infection for these shark clients are unlikely to replicate those of clients in the cleaner-reef teleost system, who become parasitized by gnanthiid isopods mostly at night while they are stationary near the substratum [33]. Yet, similar to the reef teleost system, observations of cleaner-thresher shark interactions occurred more frequently earlier in the day.

Studies have shown that cleaning frequency peaks in the early morning [12] when cleaner's guts are empty [34]. A subsequent effect of cleaners feeding at higher rates when they are hungry may be an increase in the probability of thresher sharks receiving a higher quality standard of service earlier in the day.

While cleaners' propensity for inspecting thresher sharks did not appear to be affected by shark sex or time of day, there was a direct correlation between the amount of time a shark client spent at a cleaning station and the number of inspections it received. Inspection rates and cleaning event time have been accepted as proxies for parasite infestation in previous studies [35]. Those thresher sharks, which spend more time at a cleaning station, may harbor greater abundances of ectoparasites.

Of the 19 interrupted events, 12 involved thresher shark clients sharing a cleaning station with another elasmobranch, suggesting that interactions with cleaners may be an adaptive mutualism common across elasmobranch taxa. However, relating the effects of these interactions to thresher shark health and fitness was not quantifiable and thus remains highly speculative. The rest of the interrupted events involved the arrival of SCUBA divers. While it is possible that thresher sharks interact less with cleaner fish in the presence of large numbers of SCUBA divers, inferences of human interference are treated cautiously since our camera deployment effort was nominal during peak diving industry hours (Figure 3).

### Distribution of Inspections

Preferential selection of patches by cleaner fish on a client's body was first documented from *in situ* studies in Aldabra by Potts in 1973 [36]. This early work quantified inspections by patch and inferred that cleaning strategies differ by client species. It concluded that when predatory clients interact with cleaners, the caudal fin is inspected first and the head is largely avoided. Subsequent experimental work by Bshary and Grutter showed that cleaners preferentially forage on client patches that are more heavily parasitized and that some prey items are selected over others [25], [29], [37].

Here, cleaner fish inspections on different parts of a thresher shark's body were patch specific, suggesting that the cleaners may be foraging selectively. Different areas of a shark's body are known to harbor different abundances and types of ectoparasites [11], [14]-[17], [26]. These areas may therefore provide different qualities of food patches for cleaners [25], [29], [37].

**Figure 5:**
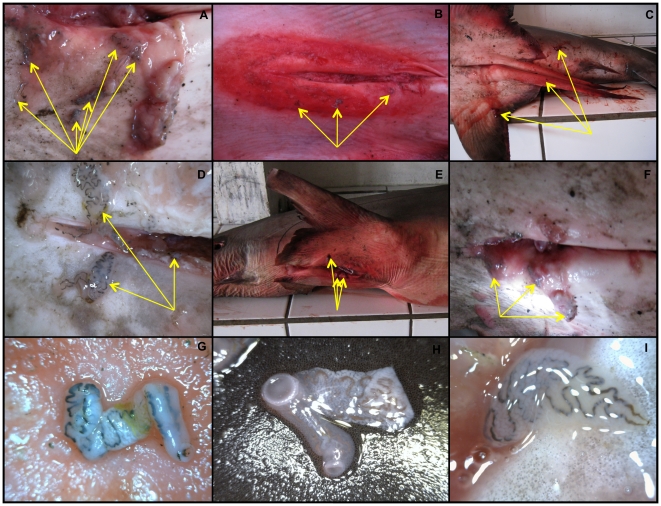
Ectoparasite infection observed in the pelvis of dead pelagic thresher shark specimens. A) Female (303 cm TL); B) Female (290 cm TL); C) Male (271 cm TL); D) Female (253 cm TL); E) Female (308 cm TL); F) Female (247 cm TL); G–I) Unidentified monogenea flatworm (Phylum Platyhelminthes) found attached in and around the cloaca of all the dead pelagic thresher shark specimens that were examined (tips of arrows in A–F).

Aggregations of large (>1 cm) unidentified monogenea flatworms (Phylum Platyhelminthes) were found attached externally around the cloaca of 11 dead thresher shark specimens taken from Philippine fish markets (Figure 5). Colonies of *Nemesis robusta* (Van Beneden, 1851), a copepod that commonly parasitizes the gills of oceanic sharks [15], were also observed in all of the sampled specimens, attached at the free distal tips of the gill filaments. These were protected by being encapsulated in the gill slits. No parasites were found elsewhere on the dead sharks. It was not possible to verify whether thresher sharks visiting Monad Shoal were comparably infected, but infection was consistent on the dead samples. It is therefore plausible that thresher sharks visiting the seamount might be similarly parasitized.

Cleaners showed significant preference for inspecting the pelvis. The monogenea flatworms believed to be abundant in this area (Figure 5), may have provided a high quality food patch for cleaners. Recent experimental studies have demonstrated that cleaners forage more on mucus and monogenea flatworms than other prey items and that they will select the latter on a size-ranked basis [29], [37]. Because of the relative ease with which cleaners can search, handle and consume large monogenea flatworms from the pelvis, it is likely that substantial cost/benefit rewards are incurred by preferentially foraging in this area. That the dorsal fin and gills were not preferred, may be due to an absence of prey items, the requirement of traveling relatively longer distances to carry out an inspection, or in the case of the gill patch, that prey items are encapsulated and thus difficult to access, and/or that they are hidden from view.

Since no ectoparasites were observed on the head and body, or the dorsal, caudal and pectoral fins of dead specimens, cleaner foraging behavior in these patches was less interpretable. Handling of the dead specimens on their way to market might have removed ectoparasites from these areas before they could be assessed. Cleaners interacting with thresher sharks may also remove mucus and/or dead tissue; one thresher shark had sustained a noticeable injury to its left pectoral fin. Since there is evidence that wound healing plays a role in cleaning services for reef teleosts [38], it is plausible that such a mechanism may also play a part in the cleaner-thresher shark association.

### Circular-Stance-Swimming

The classic head-stand or tail-stand posing behavior of reef fish clients at cleaning stations is known as a distinctive signal to solicit a cleaning interaction [20]. As ectoparasite load is known to affect a client's desire to seek a cleaner [21] and client posing rates have been related to client ectoparasite load, this willingness to pose may be explained by the increase in the probability of a client being cleaned [20].

Certain carcharhinid sharks are noted for their ability to pump their gills and for their bottom resting behavior [31], [39]. Sazima and Moura [40] described the Caribbean reef shark (*Carcharhinus perezi)* posing by lying on its side while being cleaned by the yellownose goby (*Elacatinus randalli)*. In contrast, the thresher shark, like many oceanic sharks, is an obligate ram ventilator [7], [31]. The inability to pump their gills means that pelagic thresher sharks must perpetually swim to maintain O_2_ ventilation. Thus the stereotypical immobile posing behavior exhibited by reef fish clients or that described by Sazima and Moura [40] is not possible. ‘Circular-stance-swimming’ may be an adaptation of the conventional cleaner-reef teleost system in which head and tail standing is used to pose and solicit cleaning services. A similar behavior was observed whilst researchers were measuring the swim speeds of two captive bull sharks (*Carcharhinus leucas*), which are also obligate ram ventilators [41], [42]. As cleaner wrasses approached, the bull shark was reported to slow from its routine swim speed, and assume a “head-up swimming attitude in which the longitudinal body axis was approximately 45 degrees to the horizontal” [42].

As suggested by Coté *et al.* [20] the costs and benefits of posing should be considered from both the cleaner and client's perspectives. Parasite loads and body size have been suggested as factors that may increase client ‘attractiveness’ to cleaners [35]. A moving client, which swims relatively high above the substratum, requires a greater energetic outlay to be reached by a cleaner, and therefore the thresher shark may be less attractive than a client that is sessile and close to the cleaning station. The decrease in the thresher shark's swim speed combined with the conspicuous lowering of the caudal fin and its systematic circling behavior, may provide an increased opportunity for the cleaners to inspect, thereby making pelagic thresher sharks more attractive clients.

### Seamounts and Cleaning Ecology

Evidence is mounting that seamounts support cleaning stations, which attract sharks. Scalloped hammerheads (*Sphyrna lewini*), and grey reef sharks (*Charcarhinus amblyrhynchos*) have been documented interacting with cleaners at seamounts in Costa Rican waters, and Australia respectively [43]. Previous studies have shown the importance of *L. dimidiatus* for sustaining species diversity and abundance on patch reefs [44], [45]. Visiting (non-resident) species diversity halved and their abundance fell by a quarter at Lizard Island on the Great Barrier Reef eighteen months after *L. dimidiatus* was experimentally and naturally removed [44]. At Ras Mohammed National Park in Egypt, Bshary showed a decrease in the diversity of visiting clients of ∼30% and ∼40% after similar removals of *L. dimidiatus* were conducted [45]. There, it was also found that the re-introduction of *L. dimidiatus* led to an increase of visiting species diversity by 50 to 100%. It is likely that the cleaner wrasse on Monad Shoal are equally important for structuring the community of its visiting species, and that thresher sharks visit the site to solicit their services.

### Conclusions

Our results suggest that cleaner wrasse play an important ecological role in structuring visiting elasmobranch communities at some tropical seamounts. Pelagic thresher sharks regularly visit Monad Shoal where they modify their behavior, presumably to facilitate interactions with cleaners, which may make them more attractive clients. Cleaners' selective foraging on pelagic thresher sharks demonstrates a level of preference for areas of a shark's body where specific types of parasites are found. The gradual decline in the frequency of pelagic thresher shark cleaning events from morning until evening may be driven by hungry cleaners, which provide higher quality services early on in the day. It is likely that some pelagic thresher sharks harbor greater abundances of parasites than others. Future identification and quantification of parasite loads on pelagic thresher sharks would provide further evidence that elasmobranch clients provide high quality food patches for cleaners at seamounts.

## Supporting Information

Table S1 Mean ± standard deviations (sd) of the number of recreational divers and dive boats that visited Monad Shoal, by time of day, during 232 days of field observations July 2005–December 2009.
(0.03 MB DOC)

Table S2 Matrix of post hoc analysis for estimated cleaner inspections between patches. t-tests were conducted among the estimated proportions of inspections per patch (log(pi/p1) for patch surface areas (log(hi/h1)). Test scores (t) and significance values (p) are presented with their lower (L CI) and upper confidence intervals (U CI). Cleaner preferences for patches were ranked as highly preferred (pelvis), preferred (pectoral and caudal fins), less preferred (head and body) and not preferred (gills and dorsal fin).
(0.04 MB DOC)

Video S1 A segment of an uninterrupted event, which resulted in cleaning interactions. Recorded by remote video camera 10 March 2009, on Station A, at 08:04 hours, a male pelagic thresher shark (*Alopias pelagicus*) modified its behavior by slowly circling over cleaner territories while lowering its caudal fin in a pose, presumably to facilitate cleaner inspections. This behavioral sequence, which was consistent and repetitive, was categorized as ‘circular-stance-swimming’. Note the fishing line hooked into the shark's right pectoral fin. Many of the elasmobranchs, which visit this site, are similarly affected by human activity.
(8.53 MB MOV)

Video S2 A segment of an event, which resulted in cleaning interactions being interrupted by another thresher shark. Recorded by remote video camera 10 May 2008, on Station A, at 11:03 hours, a pelagic thresher shark interacted with cleaners during eight circular-stance-swim segments before being joined by another. The two circled the cleaning station for an additional four segments before the noise of a boat propeller interrupted their behavior. Both broke from their respective circular-stance-swimming paths and left the station rapidly in opposite directions.
(9.71 MB MOV)

Video S3 A segment of an event, which resulted in cleaning interactions being interrupted by a grey reef shark (*Carcharhinus amblyrhynchos*). Recorded by remote video camera 10 November 2009, on Station A, at 09:38 hours, a female pelagic thresher shark was joined by a grey reef shark 18 circular-stance-swim segments into its cleaning event. The two sharks shared the cleaning station for one additional segment before the thresher shark left the area. Of the 19 interrupted events, 12 involved thresher shark clients sharing a cleaning station with another elasmobranch, suggesting that interactions with cleaners may be an adaptive mutualism common across elasmobranch taxa.
(7.15 MB MOV)

Video S4 A segment of an event, which resulted in cleaning interactions being interrupted by a giant manta ray (*Manta birostris*). Recorded by remote video 22 September 2005, on Station A, at 10:23 hours, a pelagic thresher shark interacted with cleaners over four circular-stance-swim segments before the arrival of a giant (∼2.5 m wingspan) manta ray interrupted its swim patterns in the fifth segment, causing it to leave the station. After five seconds, the shark returned and the two animals interacted with cleaners separately, over different areas of the same station, for an additional two segments.
(9.14 MB MOV)

Video S5 A segment of an event, which resulted in cleaning interactions being interrupted by a devil ray (*Mobula spp.*). Recorded by remote video camera 27 June 2008, on Station A, at 12:32 hours, a female pelagic thresher shark was joined by a devil ray six segments into its cleaning event. The two elasmobranchs shared the station for one additional segment before the devil ray left the area.
(9.44 MB MOV)

Video S6 A segment of an event, which resulted in cleaning interactions being interrupted by recreational SCUBA divers. Recorded by remote video camera, 08 December 2009, on Station A, at 08:16 hours, a thresher shark interacted with cleaners for 22 circular-stance-swim segments before the arrival of a group of divers interrupted its behavior. The shark abruptly adjusted its swim path and swam away to open water. Note the sound of divers alerting each other to the presence of the shark at the station in the background (vocally and by rattling on their air cylinders). It is possible that thresher sharks interact less with cleaner fish at this site, in the presence of large numbers of SCUBA divers.
(9.36 MB MOV)
