# Supplementary material for: Oceanic Sharks Clean at Coastal Seamount
Source: PLoS One. 2011 Mar 14;6(3):e14755. doi: 10.1371/journal.pone.0014755 (PMC3056710; doi:10.1371/journal.pone.0014755)
Supplement: Table S1 — Mean ± standard deviations (sd) of the number of recreational divers and dive boats that visited Monad Shoal, by time of day, during 232 days of field observations July 2005–December 2009. (0.03 MB DOC) [file pone.0014755.s001.doc]

| ***Time***  ***(hrs)*** | ***Mean Number of Divers (± sd)*** | ***Mean Number of Boats (± sd)*** |
| --- | --- | --- |
| 06:00 – 07:00 | 43.55 ± 18.71 | 5.45 ± 2.21 |
| 07:00 – 08:00 | 37.82 ± 16.86 | 5.41 ± 2.01 |
| 08:00 – 09:00 | 2.07 ± 2.07 | 1.06 ± 0.24 |
| 09:00 – 10:00 | 1.66 ± 2.11 | 1.03 ± 0.18 |
| 10:00 – 11:00 | 2.41 ± 2.41 | 1.05 ± 0.22 |
| 11:00 – 12:00 | 2.63 ± 2.57 | 1.05 ± 0.23 |
| 12:00 – 13:00 | 7.25 ± 3.92 | 1.07 ± 0.33 |
| 13:00 – 14:00 | 0.67 ± 1.49 | 1.06 ± 0.24 |
| 14:00 – 15:00 | 31.88 ± 18.64 | 4.42 ± 2.04 |
| 15:00 – 16:00 | 21.35 ± 16.44 | 3.39 ± 1.49 |
|  |  |  |
