# Supplementary material for: Oceanic Sharks Clean at Coastal Seamount
Source: PLoS One. 2011 Mar 14;6(3):e14755. doi: 10.1371/journal.pone.0014755 (PMC3056710; doi:10.1371/journal.pone.0014755)
Supplement: Table S2 — Matrix of post hoc analysis for estimated cleaner inspections between patches. t-tests were conducted among the estimated proportions of inspections per patch (log(pi/p1) for patch surface areas (log(hi/h1)). Test scores (t) and significance values (p) are presented with their lower (L CI) and upper confidence intervals (U CI). Cleaner preferences for patches were ranked as highly preferred (pelvis), preferred (pectoral and caudal fins), less preferred (head and body) and not preferred (gills and dorsal fin). (0.04 MB DOC) [file pone.0014755.s002.doc]

|  | **Body** | | **Caudal** | | **Dorsal** | | **Gills** | | **Head** | | **Pectoral** | | **Pelvis** | |
| --- | --- | --- | --- | --- | --- | --- | --- | --- | --- | --- | --- | --- | --- | --- |
|  |  | | ***t*** | ***p*** | ***t*** | ***p*** | ***T*** | ***p*** | ***t*** | ***p*** | ***t*** | ***P*** | ***t*** | ***p*** |
| **Body** |  | | 18.63003932 | **<0.001** | -1.400544601 | 0.220 | -2.650362 | **0.008** | 2.570785931 | 0.050 | 22.02273979 | **<0.001** | 33.03868402 | **<0.001** |
| **Caudal** | 0.7951 | 1.1003 |  |  | 12.07736956 | **<0.001** | 11.40527806 | **<0.001** | 15.10396611 | **<0.001** | -4.724268617 | **0.005** | -19.10637052 | **<0.001** |
| **Dorsal** | -1.7358 | -1.0542 | 2.0705 | 2.6149 |  |  | 1.037242981 | 0.347 | -3.268314475 | 0.022 | -14.06611742 | **<0.001** | -21.54581147 | **<0.001** |
| **Gills** | -2.123 | -1.443 | 2.4053 | 3.0561 | -0.0206 | 0.7966 |  |  | -4.026343827 | **0.010** | -13.05899991 | **<0.001** | -17.6967081 | **<0.001** |
| **Head** | -0.219 | 0.1506 | 0.8273 | 1.1356 | -1.6522 | -1.0694 | -2.0906 | -1.407 |  |  | -18.67168393 | **<0.001** | -29.36233484 | **<0.001** |
| **Pectoral** | 0.8405 | 1.1441 | -0.1574 | 0.0682 | -2.6589 | -2.1157 | -3.1003 | -2.4503 | -1.1801 | -0.8729 |  |  | -14.1731485 | **<0.001** |
| **Pelvis** | 1.222 | 1.5124 | -0.5235 | -0.3155 | -3.0302 | -2.4942 | -3.4722 | -2.8282 | -1.5486 | 1.2542 | -0.4775 | -0.2723 |  |  |
|  | ***L CI*** | ***U CI*** | ***L CI*** | ***U Ci*** | ***L CI*** | ***U Ci*** | ***L CI*** | ***U Ci*** | ***L CI*** | ***U Ci*** | ***L CI*** | ***U Ci*** |  |  |
|  |  |  |  |  |  |  |  |  |  |  |  |  |  |  |
